# RETRACTED: Prevalence of Histopathologically Diagnosed Opthalmic Neoplastic Lesion Among Opthalmic Biposies in a Pathology Laboratory of a Tertiary Care Hospital

**DOI:** 10.31729/jnma.7076

**Published:** 2021-08-31

**Authors:** Journal of Nepal Medical Association

**Affiliations:** 1 Journal of Nepal Medical Association, NMA Building, Siddhi Sadan P.O. Box 189 Exhibition Road, Kathmandu, Nepal

## RETRACTION NOTE

The Journal of Nepal Medical Association (JNMA) hereby retracts JNMA article,^1^ published in the year 2019, vol 57, issue 219 with page number 352-6, following the recommendations of the JNMA in-vestigation report. An official JNMA investigation team was set up following post-publication com-plaints regarding data integrity. The investigation was conducted according to the COPE retraction guideline. The authors failed to provide evidence and a satisfactory explanation. These issues could not have been detected during peer review had there been no complaint.
